# Metabolic Profile and Myocardial Performance of Renal Transplant Recipients Participating in Unsupervised Physical Exercise as a Prescription Program

**DOI:** 10.3390/jfmk3030046

**Published:** 2018-09-11

**Authors:** Luciano Moscarelli, Francesco Sofi, Gabriele Mascherini, Vittorio Bini, Chiara Ingletto, Marco Mandoli, Giorgio Galanti, Laura Stefani

**Affiliations:** 1 Nephrology Unit, University Hospital Careggi, 50139 Florence, Italy; 2 Department of Experimental and Clinical Medicine, Unit of Clinical Nutrition, University of Florence, 50139 Florence, Italy; 3 Sports Medicine Center-Clinical and Experimental Department-University of Florence, 501039 Florence, Italy; 4 Internal Medicine, University of Perugia, 06100 Perugia, Italy

**Keywords:** metabolic profile, renal organ transplantation, physical exercise, home based exercise

## Abstract

Introduction: Renal transplant recipients (RTRs) are at high cardiovascular risk (CV) compared to the general population, especially after surgical treatment. The literature supports the role of supervised exercise intervention; however no data are available regarding the effects of unsupervised exercise programs. We investigated whether a home exercise program could reduce CV risk in RTR based on possible changes in renal and cardiometabolic parameters and myocardial performance measured by echocardiography. Methods: From a large cohort of 60 RTRs, 30 RTRs (12 females and 18 males 48.3 ± 12.3 years) participated in individualized and unsupervised training programs for 6 months, at moderate intensity. Cardiometabolic risk factors, anthropometric parameters, lipid and glycemic blood sample profiles were studied as was myocardial performance from the 2D echo examination at T0, and T6 months. Results: The lipid profile remained in the range of a low level of risk, although there was no significant improvement, whereas myocardial performance, in particular the EF, was significantly improved. Conclusions: A home exercise program for at least 6 months produces positive effects on myocardial function and helps maintain a low cardiovascular risk profile. The trend supports the importance of highlighting the role of a correct reconditioning of lifestyle in RTR, from the exercise program without supervision to moderate intensity, where well tolerated.

## 1. Introduction

Renal transplantation is the treatment for end-stage renal disease [1]. It often allows the return to a normal lifestyle and participates in the survival of patients. Despite surgical treatment, patients still suffer from much higher cardiovascular morbidity and mortality than the general population [2]. Many factors contribute to increased cardiovascular mortality and of these sedentary lifestyle, hypertension, and diabetes with associated cardiometabolic risk factors are the most important [3]. In these patients, steroid use, suboptimal renal function, fat mass, and body water gain that often follows kidney transplantation can accelerate atherosclerosis [4]. Regular exercise at moderate intensity has been shown to improve the health of the general population and is also recommended for people with complex chronic diseases [5] such as RTR [6,7,8]. The authors have shown that physical activity in the post-transplant period may show low adherence [9]. The studies investigated the overall positive role of supervised exercise in RTR, while few data are available in the case of untrained and home-based training programs in this context. Especially in the population following an unsupervised program, the early diagnosis of cardiac dysfunction is justified in order to provide safe and appropriate therapeutic strategies. 2D echocardiographic myocardium is recognized to identify subclinical ventricular dysfunction that may evolve into potential cardiomyopathies [10]. The study aimed to evaluate the cardiometabolic profile and myocardial function in a group of RTRs compared to a group of control subjects (CS), participating in the same exercise program without supervision and followed for 6 months.

## 2. Methods

### 2.1. Study Population

Since 2014, from a vast cohort of 60 RTR subjects following a lifestyle reconditioning program aimed at increasing cardiorespiratory fitness, with an ideal body weight and healthy eating habits, a group of 32 subjects with and without a complete follow-up of 6 months was selected for the present investigation. Subjects with acute coronary events, arrhythmias, atrial fibrillation, or tachyarrhythmia in the previous three months were also excluded, as well subjects with uncontrolled diabetes or hypertension. Only participants of the RTR study in stable clinical condition, which completed a minimum of 6 months of the home exercise program and who returned for the next test, were considered for the study. Two of these were excluded due to the presence of an atrioventricular block despite being asymptomatic, and one was excluded for damage to the tendon which occurred during a transient pharmacological treatment with quinolon, prescribed for a urinary infection.

The remaining 30 subjects completed the evaluation. Among the RTRs, 28 had received a kidney from cadavers and two from living donors. The causes of kidney disease were chronic glomerulonephritis in 24, one polycystic kidney disease, one gestosis, one vesicoureteral reflux, one agenesis and renal dysplasia, and two undefined proteinuric renal diseases. The study, approved by the local ethics committee at the University of Florence, Italy, is a part of the national trial registered in the ISRCTN registry 20/12/2016 (Trial ID: ISRCTN66295470). All subjects gave their informed consent for participation; the study was conducted in accordance with the standards consistent with the revised Helsinki Declaration. At the beginning of the study the mean transplantation time was 10.2 ± 1.2 years. As expected, most patients had mild or moderate hypertension and all were on antihypertensive therapy (calcium channel blockers, alpha-ACE inhibitors, or ARBs). Blood pressure was generally well controlled by therapy. Immunosuppressive therapy included a calcineurin inhibitor (ciclosporin or tacrolimus) in combination with mycophenolate or everolimus and steroids (methylprednisolone). All RTRs were asymptomatic for coronary artery disease (CAD), chest pain, or arrhythmia and no evidence of uncontrolled diabetes was observed before the study began and at the start of the study. As a control group, some healthy subjects were selected. They have been tested as part of their routine health care. A complete medical history was obtained for all participants and the body mass index in height and weight (BMI, weight × height^−2^ [kg·m^−2^]) was recorded and calculated. For both groups, 2D echocardiography was performed. This is to rule out any potential cardiac contraindication to follow the exercise program and to detect initial cardiac dysfunction. Subjects were then prescribed a home-based training program without supervision with a follow-up evaluation every 6 months. Between 2014 and 2016, 23 controls (CS) were enrolled in this protocol and addressed to our sports medicine department.

### 2.2. Exercise Program

Personalized training programs were created based on the patient’s initial assessment, which included gradual ergometric tests, grip dynamometers, and chair support tests. All participants (RTR and CS) completed a top-up cycle (Ergoline 200 GmbH-Esaote) ergometric test (EMT), consisting of an incremental protocol of 25 W every two minutes according to the guidelines of the American Heart Association [11]. The hemodynamic response (blood pressure) to exercise was recorded together with heart rate (HR), power (W), and perceived strain (CR-10) according to the American Heart Association guidelines [12]. The criteria for discontinuation of the graded assessment test included voluntary exhaustion and/or the ECG criterion based on the ACSM and American College of Cardiology Association standards and/or when the subject reached at least 90% of the maximum expected heart rate for age [13]. The program was based on aerobic and strength training according to the guidelines of the American College of Sports Medicine (ACSM). The minimum goals of aerobic exercise included the achievement of 30 min of exercise, at least 5 times a week and consisted of a fast walk at moderate intensity. The intensity of the aerobic exercise was established up to 75% of the maximum effort and the corresponding level of the CR-10 effort [14] determined during the EMT.

The exercise program consisted of group and home training and with the goal of reaching 150 min·wk^−1^ of moderate exercise. Strength exercises covered at least eight muscle groups as reported in a previously suggested project for different types of diseases [8]. Only the RTR participated in a short period of aerobic exercise and supervised force, three times a week, in order to provide initial support on the correct training mode. This specific model was promoted only in the case of subjects with a history of prolonged sedentariness, as in the case of RTR. This is why the potentially well-trained controls were not included in this initial program. Thereafter, training sessions were completed at home by performing body weight exercises and using multiple aerobic conditioning modes. All exercises, established individually in accordance with the ACSM guidelines [15], have been prescribed for nonsupervised use at home (i.e., outside the gymnasium and without the presence of specialized trainers). After six months, all participants completed the follow-up visits and changes to the exercise program were made at this time. During the study there were no cardiac adverse events and the main RTR disorders were pain after completing the supervised part of the training. Adherence to the home-based program was assessed by means of regular telephone calls and also by reports written weekly in a special questionnaire, presented during the follow-up visits. Patients were asked if they were meeting minimum levels or not.

### 2.3. Metabolic Profile

All RTRs were periodically checked for their metabolic profile by measuring the blood glucose sample; creatinine, urea; total cholesterol, HDL-cholesterol, LDL-cholesterol, and triglycerides. The laboratory adopted the standard procedure for sample dosing [16]. The data were interpreted on the basis of the NCEP-ATP class, in order to describe the risk profile. Total cholesterol < 200 mg/dL; LDL cholesterol < 100 mg/dL; HDL-C > 60 mg/dL were associated with a low risk. The range of values considered normal for adults for Creatinine, Urea, and Glucose were 0.5–1.2 mg/dL, 5–20 mg/dL, and 65–110 mg/dL, respectively. Measurements were made at T0 and after 6 months at periodic medical examinations at the nephrology department of the transplant center. None of the enrolled patients followed a specific diet. However, it was recommended to follow the normal Mediterranean nutritional habits that include at least two daily portions of fruit and three of vegetables and fish three times a week and two portions of weekly cereals, as normally reported in the literature [17]. No integration was used. Adherence to this type of feeding was monitored at T0 and T6 through a special questionnaire, in which the nutritional habits of the previous week were recorded. CSs were asked to follow the same nutritional indications, however, they did not undergo a periodic control of the metabolic profile, as their lipid and glycemic profiles were normal.

### 2.4. Echocardiographic Study

Myocardial function was assessed by transthoracic echocardiography (TTE) and was performed by two expert board certified cardiologists using a MyLab™ 50 echocardiograph (Esaote, Florence, Italy) equipped with a 2.5 MHz probe. Before adding the 2D analysis of tracking Speckle Tracking (ST), cardiac function was estimated by a traditional 2D measurement of LVEF. All echocardiograms were evaluated according to the guidelines established by the American Society of Echocardiography (ASE) [12]. Systolic-diastolic rest measurements of the left ventricle (LV) included: interventricular septum (IVS), posterior wall thickness (PW), left ventricle diameter in diastolic-terminal diameter (LVEDd) and systolic-terminal diameter (LVESd), right ventricle diameter from the parasternal projection of the long axis (RV), the size of the aortic root (AOR), and the left atrium (LA). The LV cardiac mass index (CMI g·m^−2^) was calculated according to the Devereux formula [18]. As a common practice in 2D echocardiography studies, the ejection fraction (EF, %) was in the first line calculated according to the simplified formula (LVEDd-LVESd)·LVEDd-1, where the volumes are replaced by diameters. This measure was repeated in each echocardiographic assessment, then three times in the year of study for all participants. An additional measure of EF was also acquired from the volumes obtained during the deformation analysis.

From a five chamber view, the presence of valvular insufficiency was determined by continuous wave and color Doppler analysis. According to the ACC/AHA guidelines, any valvular insufficiency has been described as the extension of the regurgitation flow on a 0 to 4+ scale, using the color flow mapping method [19]. The analysis of the diastolic function was obtained from the measurements in a four-chamber view and done in the presence of a stable and repeated RR interval in three sequential measurements. The diastolic function was evaluated with Doppler measurements from the transmetric flow including the early filling peak (wave E) and late diastolic filling (wave A), isovolumetric relaxation, deceleration time, and E/A ratio. To complete the evaluation of the diastolic function, the tissue doppler was added to the speed analysis (TDI). The TDI, Ea, and Aa values were calculated as the mean of those obtained from the interventricular septum portion and the left ventricular side wall of the basal segment near the mitral valve. TDI data were compared with age-specific regulatory data, which allowed classification as normal or diastolic dysfunction [12]. For the assessment of intra-observer variability of echocardiographic data, measurements were repeated in 10 randomly selected subjects one week after the first group of calculations.

### 2.5. Statistical Analysis

All measurements are expressed as mean plus/minus standard deviation (SD). The results were analyzed using the IBM SPSS 23.0 statistical package for Windows. (SPSS Inc., Chicago, IL, USA, 2015). The Mixed-model Repeated Measures ANOVA were used to test the within subjects factor (time) and the between subjects factor (group). The paired Student *t*-test was used to analyze the metabolic variables in RTR group. A p value less than 0.05 was considered significant. A very significant value was considered with *p* < 0.001. The Pearson correlation coefficient between metabolic parameters and myocardial performance, in particular for LVEF, was also calculated.

For a good agreement among the measurements of the echo analysis, the intra-observer variability was also calculated

## 3. Results

At baseline, there were no differences in the age of RTR and CS (48.3 ± 12.3 vs. 48.0 ± 7.0), BMI were also similar (Table 1). The age at transplantation was 38.6 ± 13.1 years. None of the subjects enrolled were excluded from the study for acute cardiac events. From the questionnaire all the registered subjects declared to constantly adhere to the program are reported. Some of them have recovered the time of physical activity missed in the week with the most amount of exercise on the weekend.

At the beginning of the study, in RTR, the lipid profile was compatible with medium-low CV risk: the mean total cholesterol value was <200 mg/dL, while the LDL and HDL values were outside the normal range. The plasma levels of creatinine and urea were consistent with a previous history of renal failure (Table 1), and therefore at the upper limit of the normal range. The plasma glucose value was normal (Table 1). During the follow-up to T0, T6, the cardiometabolic parameters (total cholesterol, HDL cholesterol, LDL cholesterol, and triglycerides) as well as the parameters of renal function, creatinine, and urea, as well as glucose, did not show any significant variation (Table 1), regarding the enrollment time. Despite this, it was shown that cholesterol in particular maintained the normal range (<200 mg/dL), and therefore was around a low risk profile.

In the 28 subjects of the CS group, weight and BMI were normal for the whole study period (CST0 weight 73.4 ± 11.8 kg; CS T6 weight 71.7 ± 12.2; CS T0 BMI 23.7 ± 2, 2; CS T6 BMI 23.1 ± 2.2). Considering the normal lipid, glucidic, and renal profile of the CS, an eventual further evaluation of the blood sample during follow-up to estimate the metabolic risk was not considered in the study. The systolic and diastolic blood pressure values were normal and were maintained as normal throughout the study time in the two groups studied (Table 2).

2D echocardiographic parameters were included in the normal range for both groups and maintained throughout the study period with a good agreement among the measurements taken by the same observer. Differences were found in the echo parameters between the two groups and during follow-up. Some of them deserve to be considered, although they are shaded. We observed that the LV wall thickness values were, only in RTR, close to the upper limits of the normal range and significantly different when compared to the CS group (Table 2). This aspect could be attributed in the first line, to a presence of post-transplantation syndrome and potentially to long-term exposure of hypertension. The slight increase in thickness could be an expression of initial remodeling due to the pressure overload passed. After 6 months of exercise, these RTR parameters showed a slight tendency towards a reduction in values. This could be attributed, in the first line, to the positive impact due to the potential response to vasodilation after prolonged moderate-intensity exercise in this group. In the same group a significant progressive increase in LVEF (*p* < 0.05) was observed during the 6 months of the study. On the contrary, probably as a consequence of the moderate intensity of the prescribed exercise, in the CS group, the values showed no substantial changes during the protocol (Table 2).

A progressive reduction of the RV chamber dimension in both group (*p* < 0.001) after 6 months of exercise, despite maintaining within the normal range, was observed.

Furthermore, the LV cardiac mass index was also found higher in the RTR group and significantly different from the CS group. Although the data were not in the range of pathological cardiac hypertrophy (Table 2). As expected in those subjects over the age of 40, the diastolic parameters were at a lower level than the normal range and showed a pattern according to the data reported in the literature for the same age. Otherwise, a significant improvement, particularly in the E/A ratio, after 6 months of exercise was observed in RTR; the data reached the upper limits of the normal range at the end of the study. To complete the study of diastolic function, the measured TDI parameters, E1, and A1 waves, as an expression of the diastolic component due to the late atrial filling rate, showed higher values in the CS group, although not significantly. Data were within the normal range and without substantial differences when compared with RTR. No correlation was observed between LVEF and cholesterol data (r^2^ = 0.08). The values of systolic and diastolic blood pressure (SBP, DBP) measured to the rest are reported in Table 2. They were normal and kept normal throughout the time of the investigation, as well as the heart rate (HR) values. No significant statistical changes were observed with the exclusion of the EF of the LV chamber and RV chamber diameters

## 4. Discussion

The incidence and prevalence of cardiovascular disease (CVD) have been reported to be four to six times higher in RTR than in the general population [20]. Several factors may explain the elevated cardiovascular risk in these patients, including steroids, suboptimal renal function, weight gain, and infections. Reduced physical activity and a sedentary lifestyle are modifiable factors that could play a role in the increased mortality and morbidity of the CV [19]. It has been shown that regular physical activity is beneficial for the transplanted population. Many tests support the beneficial role of this type of intervention in other solid organs, as well as in renal transplant recipients [21,22]. Physical activity is normally prescribed in controlled conditions for this population. More recently, a home exercise program has been proposed to provide a viable alternative that can reduce excessive medicalization among all those patients in whom lifestyle refurbishment is crucial [23]. The metabolic profile in RTR is one of the most important aspects to identify a potential reduction of the CV risk factor. Conversely, a long-term exercise program could pose an additional risk to RTR. Therefore, the preliminary assessment of the impact of physical exercise, especially if suggested in an unsupervised form, is a crucial point to detect any contraindications to physical exercise.

Our study shows that the values of lipids and glucose, if they are around the normal interval, are kept within the low risk profile during at least a short period of intervention, at moderate level in RTR. Despite the intensity of the exercise being insufficient to produce significant changes to all parameters, in any case the data can confirm the importance of a correct and constant adherence to the moderate intensity training program. Despite not being monitored, none of the recruited subjects abandoned the protocol and none of them had acute events.

Above all, for myocardial performance the EF has shown significant changes as well for RV chamber dimensions that reached, at the end of the study, the lower values of the normal range. This last data will need greater evaluation in a larger cohort of subjects. In first line, it could be suggestive for a reduction of the volume overloading in the RV chamber due to the positive effects of physical exercise on the more adequate water distribution. Some other eco-parameters supported the evidence of a good result of unsupervised exercise intervention in maintaining normal values with a slight tendency toward a normalization of some specific parameters such as diastolic ones.

Noninvasive myocardial assessment remains important during the follow-up of the RTR when undergoing an exercise program. In conclusion, the results of this study confirm the positive impact of the mixed exercise program, even without supervision, in patients undergoing kidney transplantation. This protocol opens a new path aimed at giving some indications in the reconditioning of the lifestyle in RTR that seems, from the current literature, to be mostly aimed at the sedentary group after the transplantation of solid organs [24]. More attention must be given to correctly interpret the data of the possible correlation between the metabolic profile and the echo parameters. A larger sample size is required to better study this aspect, potentially related to mortality profile and not shown in this brief survey.

Some limits are therefore present in the study: the small cohort of subjects, the substantial normal profile of some parameters, at the beginning of the study, which limited any more evident changes during the protocol and the short exploration time. The small cohort in our study does not allow us to stratify patients for variables that can modify CV risk such as time spent on dialysis and time spent transplanting. As a consequence of these aspects, some other considerations are important. For example, ethnicity, gender, and the cause of renal failure examined in a larger cohort of subjects that could be more useful in the future to describe these aspects with other clinical implications. Accordingly, we cannot infer any conclusion on the need to start the exercise program for at least six months to achieve an improvement in the exercise capacity, but the data are strongly indicative.

## Figures and Tables

**Table 1:** Characteristics of renal transplant recipient (RTR).

RTR (*n* = 30)	RTR T_0_	RTR T_6_	*p* Value
Weight (kg)	70.6 ± 15.6	70.7 ± 15.3	0.830
BMI (kg/m^2^)	24.3 ± 3.8	24.9 ± 4.4	0.631
Creatinine (mg/dL)	1.5 ± 05	1.5 ± 0.7	0.265
Urea (mg/dL)	66 ± 0.26	71 ± 0.43	0.209
Cholesterol (mg/dL)	187.0 ± 55.7	194.7 ± 47.8	0.559
HDL (mg/dL)	54.9 ± 22.4	54.1 ± 16.0	0.109
LDL (mg/dL)	130.6 ± 95.0	134.5 ± 93.0	0.932
Triglycerides (mg/dL)	198.1 ± 152.4	193.3 ± 127.6	0.517
Glucose (mg/dL)	1.01 ± 0.25	0.94 ± 0.25	0.822
HbA1c (mmol/mol)	60.7 ± 11.7	57.0 ± 4.4	0.731

Legend: BMI: Body Mass Index; HDL: High Density Level; LDL: Low Density Level.

**Table 2:** Echo standard analysis and hemodynamic parameters in RTR and CS throughout the 6 months of the exercise program.

Subjects	RTR T0	CS TO	RTR T6	CS T6	ANOVA
LVDd mm	48.2 ± 3.9	48.1 ± 0.7	48.6 ± 3.6	48.5 ± 4.5	0.154
LVSd mm	29.6 ± 3.7	28.7 ± 4.3	29.4 ± 3.8	29.2 ± 3.6	0.766
IVSmm	9.8 ± 1.6 ^§^	8.7 ± 0.8 ^§^	9.8 ± 1.4 ^§^	8.8 ± 0.9 ^§^	0.187
PWmm	9.3 ± 1.1 *	8.7 ± 0.8 *	9.4 ± 1.1 *	8.7 ± 0.8 *	0.541
EF%	60.2 ± 5 ^‡^	63.6 ± 3.8	62.0 ± 3.7 ^‡^	62.4 ± 3.3	0.001
RV mm	23.3 ± 0.9 ^§^	22.1 ± 1.3 ^§^	23.2 ± 1.0 ^§^	21.8 ± 1.6 ^§^	0.001
E wave (cm·s^−1^)	72.9 ± 20.3	74.2 ± 15.0	75.7 ± 16.7	69.8 ± 13.7	0.852
A wave (cm·s^−1^)	67.4 ± 20.0 ^§^	48.1 ± 14.6 ^§^	68.3 ± 17.0 ^§^	50.2 ± 13.3 ^§^	0.501
IVRT ms	78.4 ± 14.5	75.7 ± 15.1	77.7 ± 13.5	70.0 ± 24.9	0.338
DT ms	199.8 ± 33.5	195.0 ± 36.3	183.7 ± 27.7	190.9 ± 41.6	0.530
E/A ratio	1.1 ± 0.3 ^§^	1.7 ± 0.5 ^§^	1.1 ± 0.3 ^§^	1.5 ± 0.5 ^§^	0.067
E_1_ (cm·s^−1^)	9.5 ± 3.4	10.5 ± 1.9	9.6 ± 3.4	10.5 ± 2.0	0.935
A_1_ (cm·s^−1^)	10.0 ± 2.4	11.0 ± 3.1	9.8 ± 2.5	10.7 ± 3.9	0.177
E/E_1_	8.4 ± 2.5	9.3 ± 12.8	8.5 ± 2.5	9.3 ± 13.1	0.890
LVMI gr/m^2^	104.4 ± 20.7 ^§^	88.2 ± 20.3 ^§^	109.1 ± 16.6 ^§^	90.4 ± 21.0 ^§^	0.037
SBP mmHg	130.3 ± 4.2	120.3.8 ± 4.2	125.4 ± 1.2	127.3 ± 2.2	0.564
DBP mmHg	80.2 ± 3.2	75.2 ± 3.6	78.3 ± 2.4	75.6 ± 3.1	0.233
HR	66.5 ± 5.9	68.5 ± 6.7	64.4 ± 5.4	70.64 ± 3.2	0.281

Legend: LVSd: Left Ventricle Systolic diameter; LVDd: Left Ventricle Diastolic diameter; IVS: Inter Ventricle Septum; PW: Posterior Wall; EF: Ejection Fraction; RV: Right Ventricle; IVRT: Isovolumic Relaxation Time; DT: Deceleration Time; LVMI: Left Ventricle Mass Index. E: E wave; A: A wave. SBP: Systolic Blood Pressure; DBP: Diastolic Blood Pressure; HR: Heart Rate. * Differences between RTR and CS (*p* < 0.05); ^‡^ Differences within the RTR group. ^§^ Differences between RTR and CS (*p* < 0.01).
